# Ambient Aerosol Is Physically Larger on Cloudy Days in Bondville, Illinois

**DOI:** 10.1021/acsearthspacechem.2c00207

**Published:** 2022-11-15

**Authors:** Madison M. Flesch, Amy E. Christiansen, Alyssa M. Burns, Virendra P. Ghate, Annmarie G. Carlton

**Affiliations:** Department of Chemistry, University of California, Irvine, California92697, United States

**Keywords:** aerosol optical depth, Ångström exponent, clouds, aerosol liquid water, fine particulate matter

## Abstract

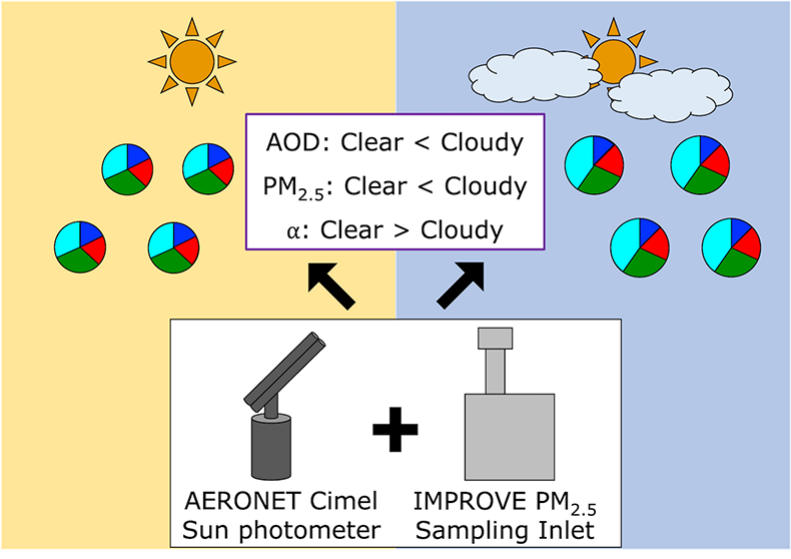

Particle chemical composition affects aerosol optical and physical properties in ways important for the fate, transport, and impact of atmospheric particulate matter. For example, hygroscopic constituents take up water to increase the physical size of a particle, which can alter the extinction properties and atmospheric lifetime. At the collocated AERosol RObotic NETwork (AERONET) and Interagency Monitoring of PROtected Visual Environments (IMPROVE) network monitoring stations in rural Bondville, Illinois, we employ a novel cloudiness determination method to compare measured aerosol physicochemical properties on predominantly cloudy and clear sky days from 2010 to 2019. On cloudy days, aerosol optical depth (AOD) is significantly higher than on clear sky days in all seasons. Measured Ångström exponents are significantly smaller on cloudy days, indicating physically larger average particle size for the sampled populations in all seasons except winter. Mass concentrations of fine particulate matter that include estimates of aerosol liquid water (ALW) are higher on cloudy days in all seasons but winter. More ALW on cloudy days is consistent with larger particle sizes inferred from Ångström exponent measurements. Aerosol chemical composition that affects hygroscopicity plays a determining impact on cloudy versus clear sky differences in AOD, Ångström exponents, and ALW. This work highlights the need for simultaneous collocated, high-time-resolution measurements of both aerosol chemical and physical properties, in particular at cloudy times when quantitative understanding of tropospheric composition is most uncertain.

## Introduction

Aerosol interactions with liquid water impact air quality and regional climate.^1^ Most aerosol mass forms in the atmosphere from reactions of gas-phase precursors and partitions into a condensed phase, including into liquid water.^2,3^ Aerosol effects on Earth’s radiation balance occur through complex mechanisms, and the magnitude of these impacts is highly uncertain.^4^ The aerosol direct effect (perturbation of solar insolation via light scattering and absorption) is a function of particle shape and size, among other variables such as particle composition and refractive index. Atmospheric liquid water uptake by hygroscopic particle-phase chemical constituents and subsequent aqueous-phase reactions has a determining effect on aerosol size.^5,6^ Liquid water efficiently scatters visible light, and its presence in the vertical column contributes to aerosol optical depth (AOD) and extinction.^7−10^ Improved air quality in recent decades over the United States, largely due to decreases in mass concentrations of the hygroscopic sulfate species in fine particulate matter (PM_2.5_—particles with an aerodynamic diameter of 2.5 μm), has helped improve visibility and reduce aerosol extinction.^11−14^ Aerosol indirect effects (perturbation of cloud properties) contribute the largest uncertainties in climate projections.^3,4^ Christiansen et al. (2020) find that PM_2.5_ chemical composition, important for overall particle hygroscopicity and water uptake, is significantly different on clear and cloudy days across the contiguous U.S. (CONUS).^15^ This suggests that accurate prediction of aerosol–cloud interactions requires quantitative understanding of aerosol properties during cloudy periods. However, this is when aerosol physicochemical properties are least understood due to a clear sky bias in atmospheric sampling. This key limitation may become increasingly problematic, in part, because global cloud cover is changing in response to climate change.^4,16,17^

Much of the chemical characterization of atmospheric aerosol in the CONUS is derived from surface networks that sample PM_2.5_ at sparsely spaced locations such as the Interagency Monitoring of PROtected Visual Environments (IMPROVE) network and the U.S. Environmental Protection Agency’s Ambient Monitoring Technology and Information Center and Chemical Speciation Network.^18,19^ The employed sampling and analysis techniques can remove aerosol liquid water (ALW)^20^ and other species during sampling and filter equilibration under laboratory conditions, which differ from the field and best characterize non-volatile particle constituents.^21^ Radiometers such as Moderate Resolution Imaging Spectroradiometer (MODIS) onboard the polar-orbiting Aqua and Terra satellites observe aerosol radiative properties unperturbed from the ambient environment and improve upon spatial and temporal resolution of surface networks. Comparison of remotely sensed aerosol radiative properties with the surface measurements of PM_2.5_ mass is restricted to clear sky conditions^22,23^ because remotely sensed observations frequently screen out measurements taken during cloudy periods due to increased uncertainty.^24−27^ Relationships between near-surface-point measurements of PM_2.5_ and columnar AOD differ by season and location.^20,28,29^ For the CONUS, correlation between PM_2.5_ and AOD in the east is generally stronger than for the west.^30,31^ The cloud-free sampling bias in AOD is a contributing factor when agreement is poor, especially in the western CONUS.^31^ Particulate nitrate, a hygroscopic constituent, is more abundant in the western CONUS and is not well captured in filter-based collection methods.^32,33^ During cloudy periods, AOD can be enhanced due to physical growth from water uptake^34−37^ and aqueous phase accretion reactions.^38^ Further, cloud processing affects the vertical profile of particulate species.^39−41^

NASA’s surface-based AERosol RObotic NETwork (AERONET) supports and provides quality assurance for satellite observations^24^ and screens final data products for cloud contamination, similar to satellite retrievals.^42,43^ The IMPROVE network shares six monitoring stations with AERONET sun photometers in the CONUS. Two were temporary AERONET campaigns and four are permanent installations for both networks. The Bondville, Illinois location, a Midwestern agricultural area, is the only site with data for both networks prior to 2013, and this area of the CONUS records suitable cloud frequency for statistical analysis year-round. AERONET computes AOD at a given wavelength from the total optical depth measured by the sun photometer at discrete spectral wavelengths from the surface to the top of the atmosphere through removal of contributions from Rayleigh scattering optical depth and spectrally dependent atmospheric trace gases.^42^ Specifically, Ångström exponents are calculated using least-squares regression of AOD for each non-polarized wavelength measured between two channels. AERONET uses the 440, 500, 675, and 870 nm channels to determine the 440–870 nm Ångström exponent. Ångström (1929) represents this spectral dependence of aerosols by a power law relationship (eq 1).^44^
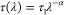
1Here, τ(λ) is the AOD at a specific wavelength λ, τ_1_ is the approximated AOD at 1 μm, and α is the Ångström exponent. From this, the Ångström exponent can be derived to qualitatively estimate the size distribution of aerosols within the vertical column.^44−46^ Literature-reported Ångström exponents often use wavelengths within the range of 440 and 870 nm, as the former is sensitive to the fine mode radius and the latter is sensitive to the fine mode volume fraction.^47^ Chemically generated fine mode particles typically have values of α ≥ 2, while physically generated coarse mode particles or evenly mixed fine and coarse mode populations have values of α ≤ 1.^45,47−49^ Several previous studies pair near-surface PM_2.5_ mass measurements with satellite-based total column AOD observations via MODIS and demonstrate statistically significant correlation during cloud-free conditions. However, aloft aerosol, when present, confounds comparison because it is not captured in point measurements at the surface.^31,50−52^ By comparing chemically speciated surface PM_2.5_ mass concentration with columnar AOD and Ångström exponent estimates, we investigate associations among physicochemical properties under clear sky and cloudy conditions.

We employ a combination of surface measurements, satellite products, and thermodynamic modeling to analyze seasonal trends at a collocated AERONET and IMPROVE network site in rural Bondville, IL that has a 10 year record for both networks and frequent cloud cover during all seasons. We capitalize on AERONET’s cloud screening process to study differences in aerosol mass, chemical composition, and optical properties as a function of sky cloudiness conditions as observed from Earth’s surface. The surface-based AERONET station is ideal for identifying low-level clouds that interact with boundary layer aerosol. We quality check this method with geostationary operational environmental satellite (GOES) observations of cloud top temperature over a representative year of data for this location. We explore seasonal trends in Ångström exponents, AOD, meteorology, and PM_2.5_ chemical composition on days that are predominantly cloudy or clear sky from 2010 to 2019 within the context of AERONET cloud flagging. We seek to infer differences in atmospheric particle size, a key determining factor in aerosol lifetime, that is attributable to plausible chemical explanation.

## Experimental Section

We analyze differences in aerosol optical properties and particle-phase species mass concentrations for a collocated AERONET and IMPROVE network monitoring location in rural Bondville, IL. We use all available surface air quality measurements from January 2010 to December 2019 from the IMPROVE network public archives for the monitoring station (40°05′20″ N, 88°37′33″ W).^18^ IMPROVE surface PM_2.5_ mass concentrations of sulfate, nitrate, organic carbon (OC), sea salt, and crustal species such as calcium, potassium, and magnesium are available as 24 h integrated samples every 3 days. Sulfate and nitrate concentrations are determined using ion chromatography, OC fractions via thermal/optical reflectance, and crustal and sea salt species are determined by X-ray fluorescence (XRF). We assume all XRF species are present as fully water-soluble concentrations in our particle water estimates, as described below, although these species may be part of non-water-soluble matrices such as silicates and dust. While this introduces some uncertainty, sulfate and nitrate largely control overall estimated ALW mass concentrations. For quality assurance, we estimate ALW mass with and without crustal species, finding that ALW estimates are insignificantly higher with these species excluded (Figure S1). AERONET public archives provide surface-based estimates of columnar AOD at 440, 500, 675, and 870 nm and Ångström exponents estimated for the 440–870 nm spectral range at Level 1.0 and Level 2.0 quality levels.^53^ We define winter as December, January, and February; spring as March, April, and May; summer as June, July, and August, and fall as September, October, and November. A cloud determination method assigns surface measurements into two bins, “predominantly cloudy day” and “predominantly clear sky day” (discussed below). We use the Mann–Whitney *U* test, a nonparametric statistical test, in R statistical software^54^ to compare two non-normal population distributions. The Mann–Whitney *U* test determines the probability that a sample from the clear sky population will be smaller or larger than a sample from the cloudy population. We determine statistical significance of *p* < 0.05 for differences in the populations for Ångström exponents, AOD, and PM_2.5_ chemical composition.

We estimate mass concentrations of particle-phase water using the inorganic thermodynamic equilibrium model ISORROPIAv2.1 in the reverse, open-system direction.^55^ We assume metastable particles with ammonium nitrate and ammonium sulfate in the aqueous phase^56^ and a well-mixed boundary layer. We estimate inorganic aerosol liquid water (ALW) using all available IMPROVE-reported mass concentrations of fine particulate matter chemical constituents. IMPROVE does not measure ammonium ion concentrations at the Bondville location, and neglecting this species in an agricultural area such as Bondville adds uncertainty to ALW estimates. Ammonia is abundant in agricultural areas and facilitates the formation of particulate nitrate, a hygroscopic species with substantial losses from filter measurements, in particular during summer.^32,33^ Seasonal cycles and temporal trends of ALW estimates are similar with and without ammonium from 2010 through 2015 for IMPROVE sites across the CONUS.^15^ At the Bondville location, seasonal average ALW estimates with weekly aggregated ammonium filter measurements included from a nearby CASTNET site^57^ are not significantly different at the 95% confidence level (Figure S2). We use daily averaged temperature and relative humidity (RH) from the European Centre for Medium range Weather Forecasting (ECMWF) reanalysis model (ERA5) meteorological outputs for estimating ALW mass concentrations.^58^ The ECMWF ERA5 reanalysis model yields hourly averages of surface temperature, dewpoint temperature, and planetary boundary layer (PBL) height. We take daily averages of both temperature values before calculating surface RH using the formula in Huang (2018).^59^ We screen RH values >95% due to constraints in ALW estimates, although no daily averages were above this threshold for the study period. As in Christiansen et al. (2019) and Nguyen et al. (2015), we estimate organic ALW using the κ-Kohler theory and the Zdanovskii–Stokes–Robinson mixing rule shown in eq 2.^60,61^
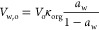
2

Briefly, we assume water activity (*a*_w_) to be equivalent to RH. *V*_o_ and *V*_w,o_ are organic matter volume and the associated water volume, respectively; κ_org_ is the assumed organic hygroscopicity parameter of 0.3 employed for rural aerosol.^15,62,63^ We use a mass balance method to calculate organic mass (OM) from IMPROVE-measured OC fractions with site- and season-specific (for each year) OM/OC ratios, as described in greater detail elsewhere.^15,64^ Note that estimated OM/OC ratios at IMPROVE sites across the CONUS including at Bondville exhibit an upward trend over the last 20 years,^64^ as do gravimetric mass measurements since 2011.^65^ We divide OM by an assumed density of 1.4 g cm^–3^ to determine *V*_o_.^66,67^

The time resolution of the various data products employed creates a limitation in this analysis. AERONET measures aerosol optical properties every 15 min during daylight hours, while IMPROVE provides 24 h integrated chemical measurements every 3 days. However, directly measured in situ ALW mass concentrations change over the course of a day in response to changing meteorology and particle chemical composition.^62^ Therefore, differences in ALW mass estimates during cloudy versus clear sky conditions described here may be different than we are able to quantitatively assess with existing data sets and limitations of current measurement techniques. This likely results in understated differences for the predominantly clear sky and predominantly cloudy days in these findings because daily averages obscure diurnal patterns in meteorology and particle hygroscopicity that have a direct impact on ALW mass concentrations on a diurnal time scale.

We assess a cloudiness classification method at Bondville, IL: interpretation of the AERONET cloud screening algorithm evaluated with the spatiotemporal pairing of GOES cloud top temperature measurements. AERONET Level 1.0 products, Level 2.0 products, and related cloud information are available approximately every 15 min. This improves upon temporal limitations associated with the MODIS Cloud Mask used in a previous analysis,^15^ which is only available once or twice daily. We match all AERONET observation days to IMPROVE measurement days. For quality assurance of the cloudy versus clear classification method, we evaluate half-hourly GOES meteorological observations from 2017, a representative year during the study period, and match to the nearest AERONET observational times within a 30 min window around the GOES observation. We derive a cloud mask from the AERONET quality assurance (QA) cloud screening algorithm to assess impacts of retrieval cloudiness category on the measured AOD and Ångström exponents.^42^

**Figure 1:**
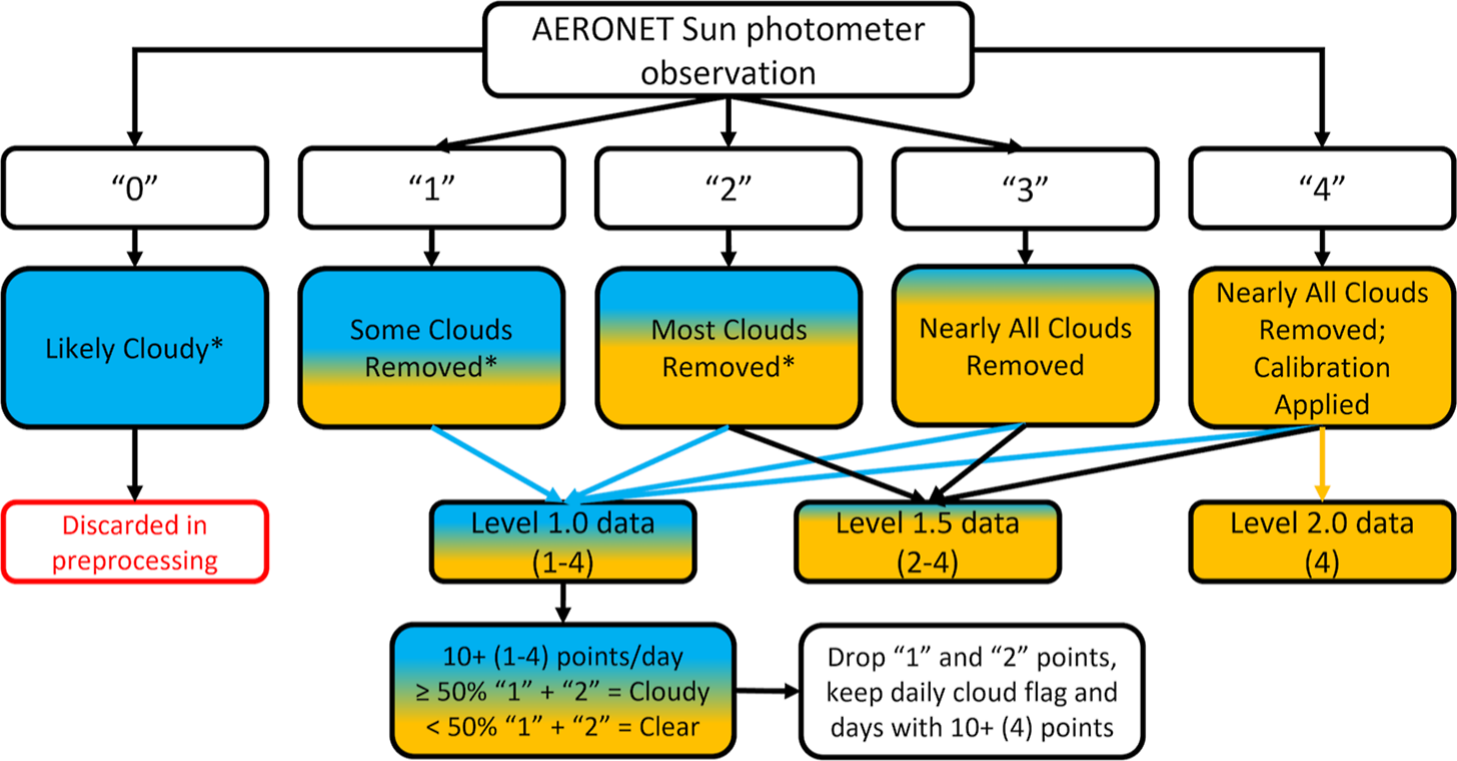
Flow chart of the cloud processing method for sun photometer observations by the AERONET quality assurance algorithm and subsequent categorization and analysis performed within this work. Note that these metadata flags are not available in AERONET public data products. Each number is indicative of the maximum achieved quality level and those demarked with * can be impacted due to instrument operation issues or measurement anomalies. With this, “0” and “1” are “cloudy”, “2” as “some clouds”, and “3” and “4” as “clear sky”.

We use the AERONET QA algorithm that identifies each observation made by the sun photometer with specific data quality metrics, provided upon request for this analysis, which are available in the associated data repository for this publication. Pre-processing removes observations due to instrument errors or full cloud obscuration of the field of view (marked as a “0” in the meta data). AERONET releases all other observations marked on a scale of 1 to 4 in their public data products, indicating the maximum quality level of the retrievals.^42^ A “1” is likely cloudy, a “2” has most clouds removed, a “3” has nearly all clouds removed, and a “4” has nearly all clouds removed and final calibration applied. For the study period at the Bondville site, no “3” observations are recorded. Level 1.0 data products retain all observations marked “1” to “4,” while Level 2.0 data products retain only those observations marked “4”. For both data products, we analyze all days with 10 or more non “0” retrievals during AERONET observational hours. We deem an individual day in the Level 1.0 product “predominantly cloudy” if the number of observations marked “1” and “2” meets or exceeds 50% of the total number of measurements for the day (Figure 1). We employ “1” and “2” (cloud-impacted) observations only to determine cloudiness classification. In the statistical comparison between cloudy and clear categories, we use days with 10 or more quality-assured “4” points only.^42^ Thus, while we use the Level 1.0 product for determining an implicit daily cloud flag, our statistical evaluation uses only observations that qualify for the Level 2.0 data product. All AOD measurements pass the triplet variability quality assurance step in the algorithm and have approximate uncertainties of 0.02.^42^ The AERONET screening algorithm uses solar aureole radiance with respect to the scattering angle to account for and remove high thin cirrus clouds, but cloud contamination for low optical depth (AOD < 2) clouds is possible.^42,68,69^ The sun photometer can find the sun during broken cloud conditions during some of the days binned as “predominantly cloudy,” and the differences presented here represent a lower boundary for distinction. For brevity, legends and figure captions will refer to the cloudiness bins defined above as “cloudy” and “clear sky”. We cross-check instrument downtime and periods of collimator tube obstructions (e.g., spider webs or water droplets), removing any retrievals that passed pre-processing steps by the QA algorithm but fit either of these criteria, as well as AOD measurements with values above 1.5 to retain only physically realistic measurements for this location. We bin observations by day to be consistent with the 24 h chemical speciation measurements from IMPROVE.

Agreement of sky conditions via this method is supported with measured cloud top temperatures from the GOES historical archive and lends support to the suitability of our AERONET-derived categorical cloud determination method.^26,27,70^ GOES observes a range of meteorological variables every half hour, and we use the cloud top temperature measurement^26,27,43^ to determine how frequently both GOES and AERONET identify clouds over the Bondville site. We sample the year 2017 and match AERONET observations from the Level 1.0 data product and pair in space and time within 15 min of the corresponding GOES measurement. For example, a GOES measurement of cloud top temperature at 12:45 PM is paired with valid AERONET observations (“1” to “4”) within the time frame of 12:30–12:59 PM. We bin each GOES observation as “predominantly cloudy” if a cloud top temperature is recorded, and “predominantly clear sky” if GOES does not observe clouds at the Bondville location. We deem a GOES-based day as “predominantly cloudy” if 50% or greater of the GOES observations record cloud top temperatures. GOES detects high-level thin cirrus clouds, but the GOES-13 lacked the 12 μm channel to detect this cloud type well.^69,71^ The spatial and temporal resolutions of the GOES data are 9 km and 30 min, respectively. It is therefore possible for GOES to miss boundary layer shallow cumulus clouds with a smaller spatial extent and shorter lifetime. In addition, cloud top temperatures are not retrieved during broken cloud conditions. GOES retrievals are not available for our entire timeframe. Direct comparison of the methods for 2017 finds that the daily predominantly cloudy or predominantly clear day bin definitions for AERONET and GOES (Figure S3) agree for approximately 76% of the paired comparisons. We rely on the AERONET method in this analysis.

## Results and Discussion

**Figure 2:**
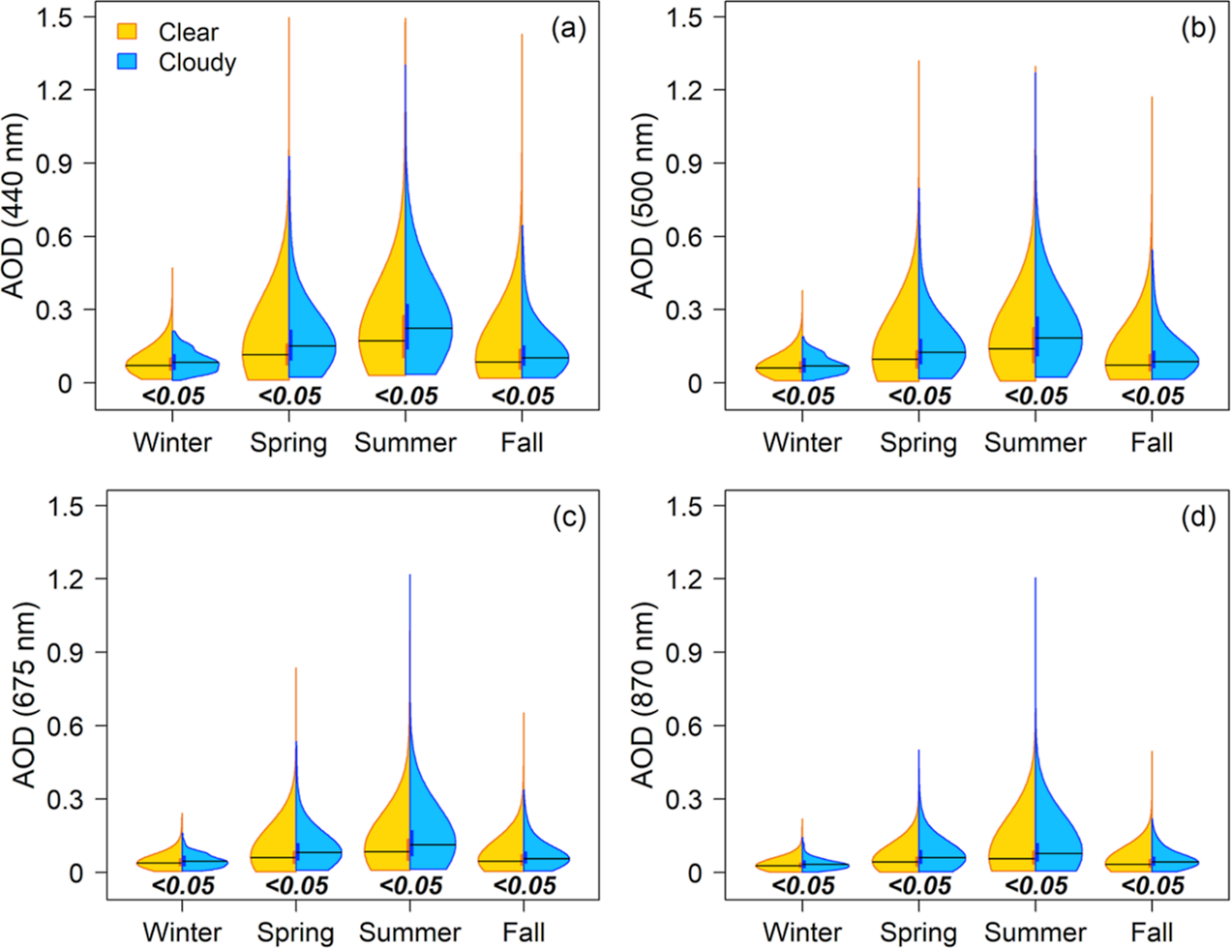
Seasonal distributions from 2010 to 2019 of AOD at (a) 440, (b) 500, (c) 675, and (d) 870 nm on clear sky (gold) and cloudy (blue) days as binned by the AERONET quality assurance algorithm method. Vertical gold (clear sky) and blue (cloudy) bars represent the 25th to 75th quartile of each distribution, with the black horizontal line as the median. Black numbers are *p*-values from two-sided Mann–Whitney *U* tests, with bold italicized font indicating statistical significance between distributions where a *p* value < 0.05 indicates the two populations are not equal.

**Figure 3:**
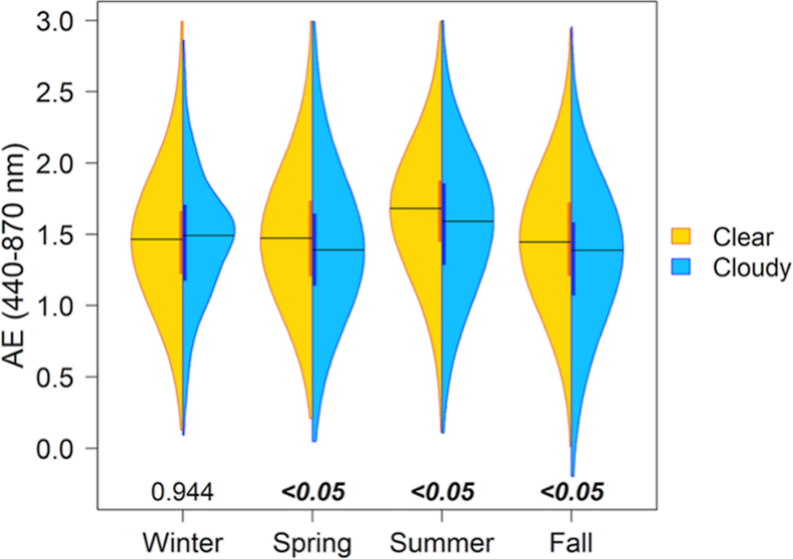
Seasonal distributions from 2010 to 2019 of AE for the 440–870 nm wavelength range on clear sky (gold) and cloudy (blue) days. Cloud bins, coloring, numbers, and statistical significance are as in Figure 2.

During the study period in Bondville, population distributions for values of AOD at all measured wavelengths of 440, 500, 675, and 870 nm are significantly different on clear sky days than on cloudy days in every season (Figure 2). On clear days, Ångström exponents (AE) are significantly larger, indicating a smaller average physical size for the ambient aerosol distribution (recall the inverse relationship in eq 1), with the sole exception of winter (Figure 3). In spring and summer, median AOD values are greatest at all examined wavelengths, and differences between cloudy and clear sky days are most pronounced (Table S1). There are more predominantly clear days than predominantly cloudy days in every season, with 16–27% of days binned as “cloudy” (Table S2). The difference in medians is small, yet more often than not, is more than the 0.02 instrumental uncertainty.

**Figure 4:**
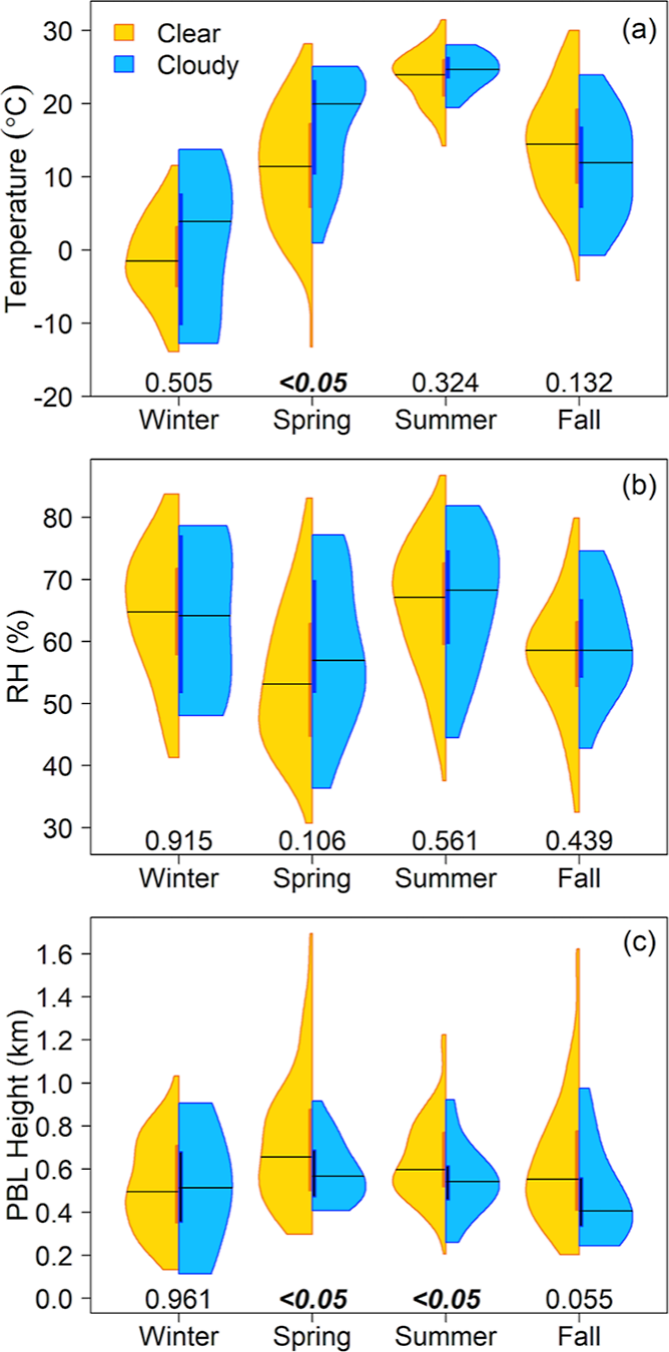
Seasonal distributions of (a) temperature, (b) relative humidity, and (c) planetary boundary layer height on clear sky (gold) and cloudy (blue) days. Cloud bins, coloring, and statistical significance are as in Figure 2.

Meteorological variables of temperature, RH, and PBL height affect AOD and are insufficient to fully explain the predominantly clear and cloudy period AOD and AE differences. Integration over the PBL height, where most aerosols reside, is a primary driver of AOD. In spring and summer, when the boundary layer height is the highest, the AOD is the greatest. However, during spring and summer, the PBL height is significantly higher on clear sky days (Figure 4), while, in contrast AOD is larger on cloudy days. While particle growth through humidification is consistent with elevated AOD and smaller AE values on predominantly cloudy days,^72^ differences in RH are not significant for any season. Temperature does not exhibit significant differences on clear sky and cloudy days in most seasons at Bondville, apart from higher temperatures on cloudy days in the spring (Table S3). Wintertime meteorology at Bondville seems to be similar regardless of cloudiness bin for all variables, although this season has the fewest cloudy days (Table S2). Surface-sensible and latent heat fluxes or large-scale subsidence could induce seasonality in meteorology, including the PBL depth, rather than the boundary layer temperature and humidity that are evaluated here. This indicates that factors other than the meteorological variables we evaluate here (i.e., *T*, RH, and PBL) may play a role in the observed differences in aerosol optical properties.

**Figure 5:**
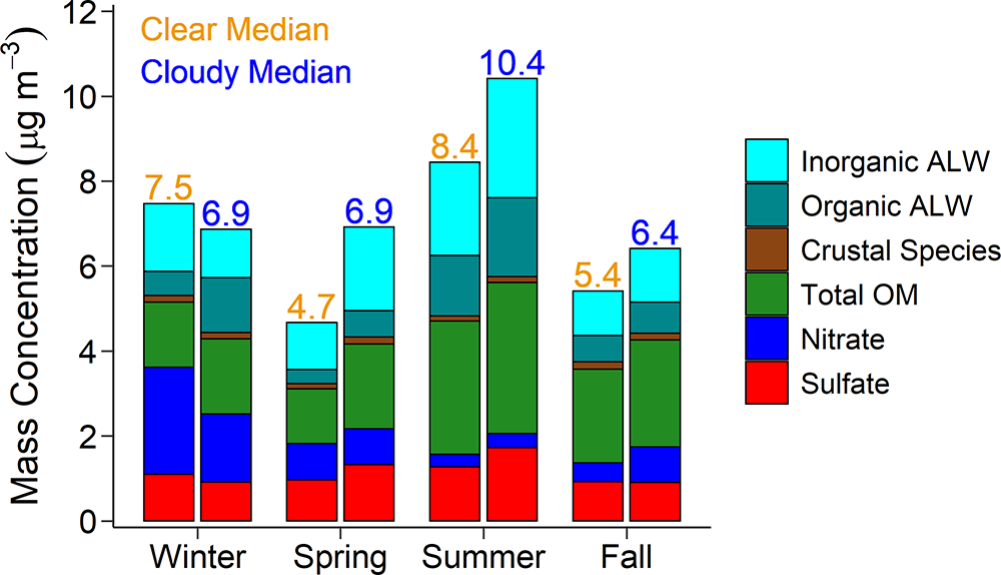
Stacked bars of median total PM_2.5_ chemical composition and ALW observation-based estimates during clear sky (left bars, gold numbers) and cloudy (right bars, blue numbers) days as binned using the AERONET QA algorithm for 2010–2019. Crustal species include calcium, potassium, magnesium, and sea salt species of sodium and chloride. Total OM was calculated from IMPROVE-measured OC using OM/OC ratios defined in the Experimental Section. Table S4 identifies statistical significance for individual chemical constituents.

Chemical composition of PM_2.5_ provides a plausible explanation for AOD and AE observations at the Bondville monitoring site, should surface measurements adequately represent the boundary layer column. Particle composition affects AOD and AE via intrinsic properties such as refractive index and through influences on particle size via changes in hygroscopicity and water uptake.^73,74^ Cloudy versus clear sky differences in overall PM_2.5_ and ALW mass are sharpest during spring and summer, exhibiting similar patterns to AOD and AE measurements for those categories. In the summer when PM_2.5_ and ALW mass are the highest, AOD is also the highest. IMPROVE-measured PM_2.5_ mass concentrations that include ALW are the highest on cloudy days in every season but winter (Figure 5). This is similar to patterns in AOD and is physically consistent with an abundance of hygroscopic particles that take up water to grow in size on cloudy days. Aqueous-phase processing of air parcels is documented to shift accumulation mode aerosol to a larger population size.^75,76^

PM_2.5_ chemical speciation determines particle hygroscopicity, water uptake, ALW mass concentrations, and subsequently aerosol size. Christiansen et al. (2020) found significant differences between cloudy and clear sky distributions of particle chemical constituents, especially ALW, using the MODIS Cloud Mask approach paired with IMPROVE PM_2.5_ chemical speciation over a continuous 5 year period across the CONUS.^15^ Springtime distributions in sulfate, ALW, and organic matter are significantly different on predominantly clear and cloudy days in Bondville. Distributions in RH for cloudy and clear sky days do not exhibit significant differences in any season and cannot be the sole explanation for clear versus cloudy patterns in ALW mass concentrations. In Bondville, sulfate concentrations are highest in summer and spring on cloudy days when ALW mass concentrations are estimated to be greatest and AOD is observed to be highest. Elevated sulfate and ALW mass on cloudy days are consistent with the hypothesis that an abundance of hygroscopic aerosol in the boundary layer can serve as cloud condensation nuclei to affect cloud systems.^77,78^ In contrast, ALW mass concentrations during wintertime are highest on clear days when nitrate is most abundant. In the Po valley, an agricultural region of Italy, nitrate was found to have a determining impact on ALW.^79^ Organic mass varies seasonally and is a key source of uncertainty. Organic species can alter intrinsic volumetric absorptive properties important for AOD, and the associated hygroscopicity is poorly understood relative to inorganic salts. The chemical composition that controls water uptake in Bondville during winter (nitrate) versus spring and summer (sulfate) may contribute to seasonal patterns regarding clear versus cloudy optical measurements of AOD and AE.

Decadal analysis indicates that sulfate and inorganic ALW mass concentrations decreased in Bondville (Figure S4), similar to trends in sulfate and ALW reported for the southeast region of the U.S.^12,61^ This is suggestive that with less water uptake, smaller particles would exhibit larger AE values over time, but there is not a clear trend in AE. This may be due to increasing organic mass, or nitrate mass, which initially declines and then dramatically increases (factor of 8) in recent years (Figure S4). Also, an increase in coarse-mode aerosol (PM_10_)—particles with aerodynamic diameters of 10 μm—that are not evaluated here, may affect the AERONET-measured AOD and the reported AE. Hand et al. (2017) and Malm et al. (2020) suggest increasing PM_10_ over the CONUS in recent years.^80,81^ The AE value on clear sky days is larger, suggesting an aerosol population more dominated by fine mode aerosol;^49^ however, this may not be observed in every circumstance.^38^ Previously in agricultural midwest locations, investigators find several factors, including organic species, control AOD, and extinction.^50,51^ Mass concentrations of OM and organic ALW (estimated from OM, not OC) do not decline over the decade. The ratio of OM/OC varies substantially by season (Figure S5), indicating substantial changes in organic constituents. Extinction properties of ambient carbon may vary as the OM/OC ratio does and this may also influence these trends.^64^ A higher OM/OC ratio indicates more oxidized organic aerosol, which can be more hygroscopic. Over the studied decade, because sulfate mass is decreasing while OM mass is not, the fractional contribution of organic species to particle dry mass and influence on overall particle hygroscopicity is increasingly important. Critical open questions regarding water uptake by particle-phase organic species remain.

## Conclusions

During 2010–2019 at the collocated AERONET and IMPROVE network monitoring stations in rural Bondville, IL, median AOD at 440, 500, 675, and 870 nm is higher on cloudy days in every season. Ångström exponents are smaller on cloudy days in every season except winter when nitrate mass concentrations are highest. Meteorological variables of temperature, RH, and PBL height are insufficient to fully explain the statistical significance for differences in AOD, Ångström exponents, and ALW mass concentration on predominantly clear sky versus cloudy days.

Aerosol chemical composition that alters particle hygroscopicity to affect water uptake and growth is a plausible explanation consistent with observations that suggest physically larger particles and higher AOD measured by AERONET on predominantly cloudy days. Size largely determines aerosol extinction and lifetime, critical parameters that define particle impacts on air quality, regional radiation budgets, and surface temperature. Our findings here suggest that aerosol size is different on cloudy days, when tropospheric composition is least understood. This warrants further study and highlights the need for collocated chemical, optical, and physical aerosol measurements at high time and vertical resolution, including at cloudy times, when quantitative understanding of boundary layer aerosol is least robust.

## Supporting Information Available

Methodology, satellite-surface comparisons, seasonal time series for OM/OC ratios, PM_2.5_ chemical species, ALW sensitivities, Ångström exponents, and clear sky and cloudy medians and associated *p*-values from Mann–Whitney U tests (PDF)

## Notes

AERONET quality assurance algorithm data flags, associated code, and examples of statistical code are available at the associated GitHub repository https://doi.org/10.5281/zenodo.7216972.
