# Supplementary material for: Ambient Aerosol Is Physically Larger on Cloudy Days in Bondville, Illinois
Source: ACS Earth Space Chem. 2022 Nov 15;6(12):2910–8. doi: 10.1021/acsearthspacechem.2c00207 (PMC9761781; doi:10.1021/acsearthspacechem.2c00207)
Supplement: Supplementary file 1 — sp2c00207_si_001.pdf [file sp2c00207_si_001.pdf]

Supplemental Information for Ambient Aerosol is Physically Larger on Cloudy Days in Bondville, Illinois

*Madison M. Flesch, Amy E. Christiansen†, Alyssa M. Burns, Virendra P. Ghate[a], Annmarie G. Carlton**

[1]Department of Chemistry, University of California, Irvine, Irvine, California 92697, United States

† Division of Energy, Matter and Systems, University of Missouri – Kansas City, Kansas City, Missouri, 64110, United States

[a]Environmental Science Division, Argonne National Laboratory, Lemont, IL, 60439, United States

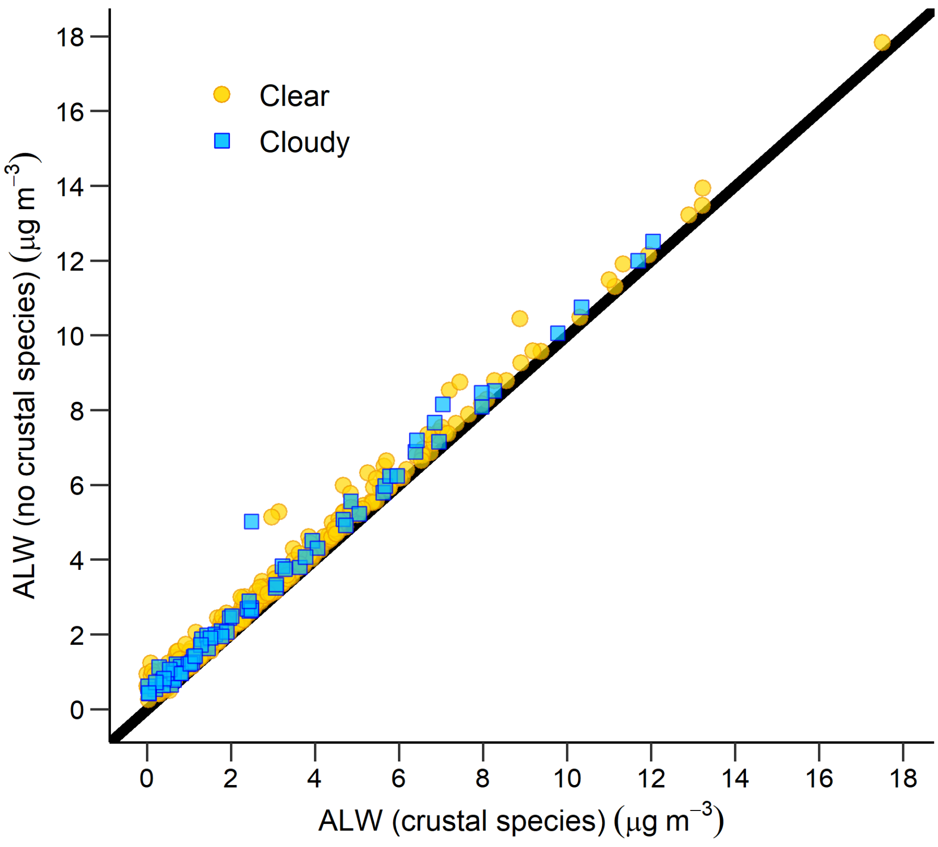

Figure S1. Comparison of ISORROPIA model outputs of aerosol liquid water (ALW) mass concentration estimates for 2010 – 2019 at Bondville on predominantly clear sky (gold circles) and predominantly cloudy (blue squares) days. Crustal species include calcium, potassium, magnesium, and sea salt constituents of sodium and chloride. Temperature, relative humidity, and mass concentrations of sulfate and nitrate were kept constant. The black line is a 1:1 line for reference. ALW estimates average 14% lower when crustal species are included.

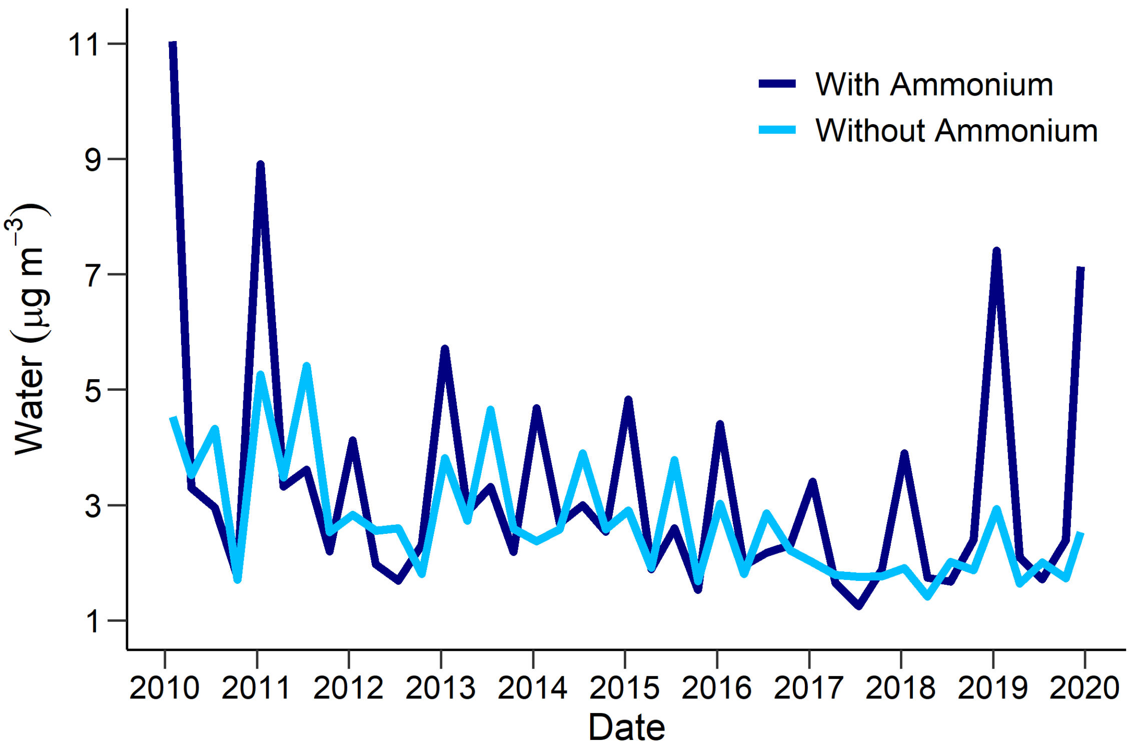

Figure S2. Comparison of seasonal average aerosol liquid water (ALW) mass concentration estimates for 2010-2019 at Bondville. Temperature, relative humidity, and mass concentrations of sulfate, nitrate, and crustal species were kept constant. Ammonium values are taken from a co-located CASTNET site, and seasonal aggregates are used as inputs to ISORROPIA. The dark blue trace includes ammonium, while the bright blue trace excludes ammonium. ALW estimates are approximately 21% lower without ammonium, though this is not statistically significant for a T-test with 95% confidence.

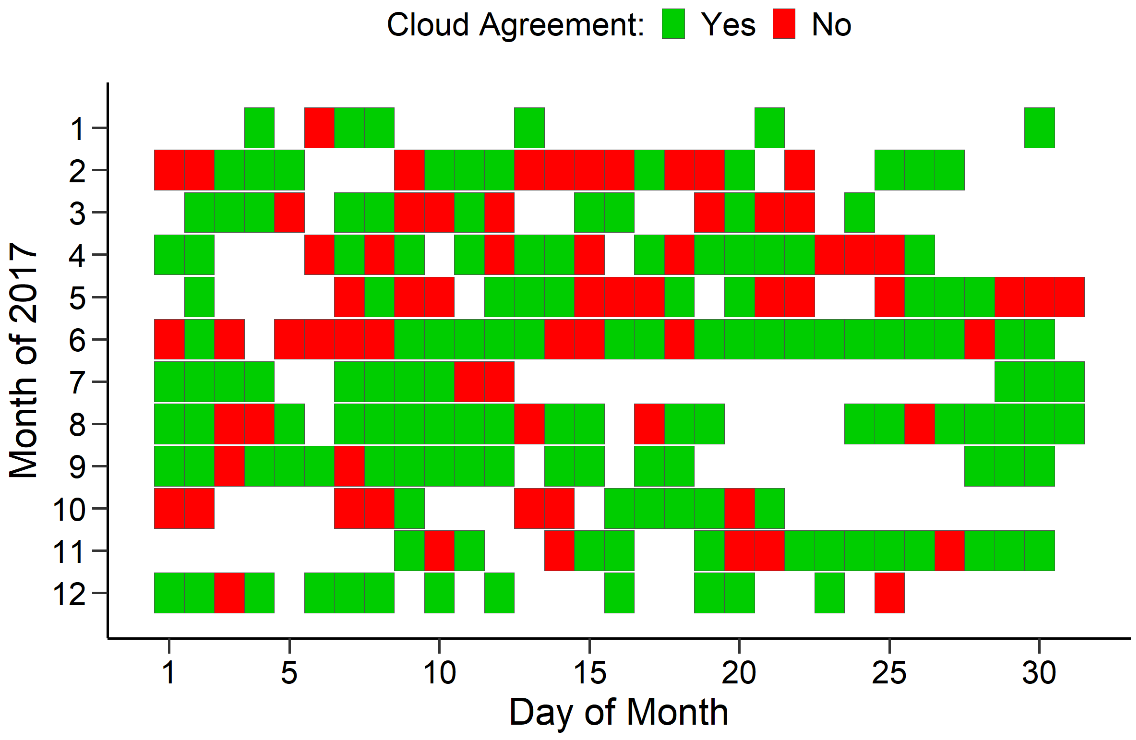

Figure S3. Comparison of the AERONET-based cloudiness determination method to GOES-measured cloud top temperature at the Bondville location for daily aggregate AERONET observations for 2017. Green indicates both methods agree, red indicates the methods disagree, with agreement occurring approximately 76% of the time. GOES-13 did not have the 12 μm channel available for thin cirrus cloud detection, which AERONET's algorithm screened better at the time, plausibly contributing to most of the disagreement occurring from AERONET measurements binned as cloudy and GOES retrievals binned as clear sky.

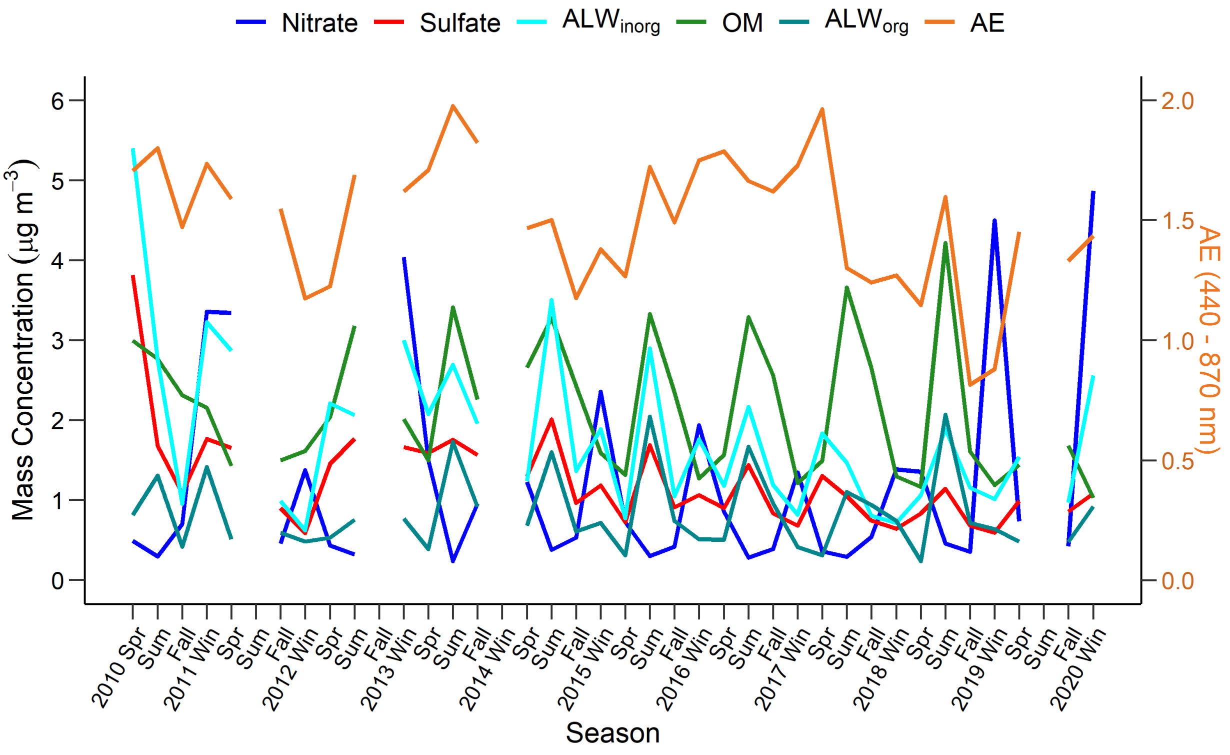

Figure S4. Angstrom exponents (orange) and mass concentrations of nitrate (blue), sulfate (red), inorganic aerosol liquid water (ALW_{inorg}, cyan) organic matter (OM, green) over time, and organic aerosol liquid water (ALW_{org}, dark turquoise) at Bondville. Gaps indicate a lack of quality assured data from AERONET for that season that was matched to IMPROVE measurement days. Note that "2020 Win" only contains data from December 2019.

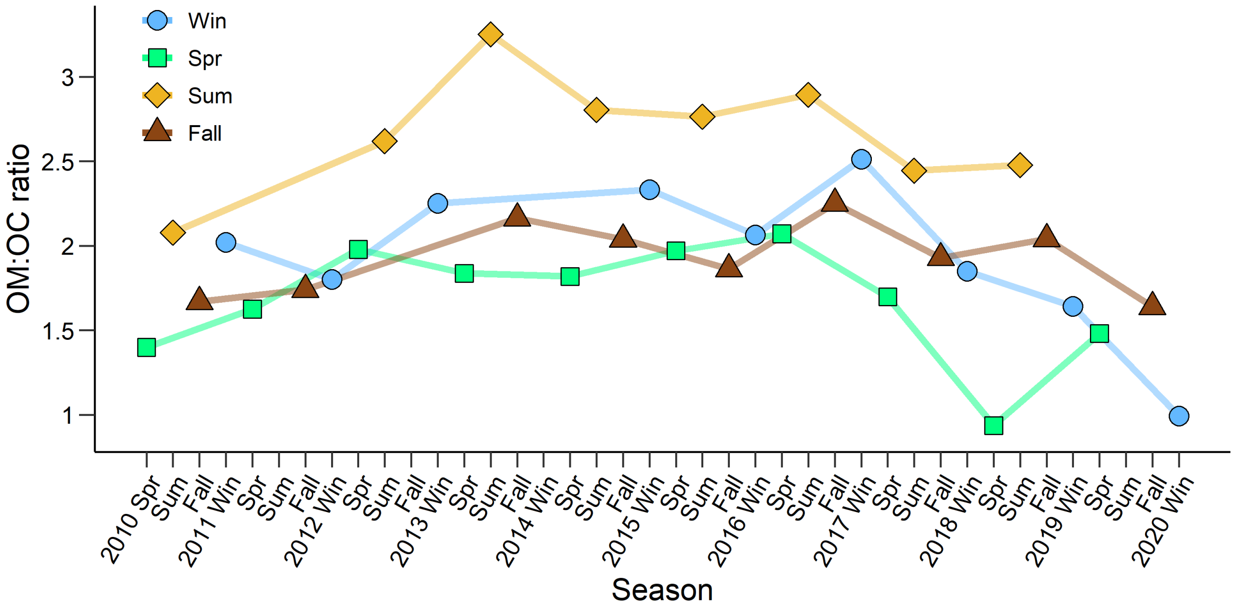

Figure S5. Time series of the ratio of organic matter (OM) to organic carbon (OC) calculated annually for winter (blue circles), spring (green squares), summer (gold diamonds), and fall (brown triangles) at Bondville. Gaps in points for each season indicate missing data for that season based on a lack of quality assured data from AERONET for that season that was matched to IMPROVE measurement days. Note that "2020 Win" only contains data from December 2019.

Table S1. Predominantly clear sky and cloudy medians and two-sided Mann-Whitney U test p values of aerosol optical depth (AOD) and Ångström exponents. For clarity, p values significantly less than 0.05 are listed as "< 0.05" and statistically significant seasons are bolded.

Parameter	Season	Clear Median	Cloudy Median	Mann-Whitney U test p value	Medians > 0.02 Experimental Uncertainty
Angstrom exponent (440 – 870 nm)	Winter	1.46	1.49	0.944	Y
	Spring	**1.47**	**1.39**	**< 0.05**	Y
	Summer	**1.68**	**1.59**	**< 0.05**	Y
	Fall	**1.45**	**1.39**	**< 0.05**	Y
Aerosol optical depth (440 nm)	Winter	**0.07**	**0.08**	**< 0.05**	N
	Spring	**0.11**	**0.15**	**< 0.05**	Y
	Summer	**0.17**	**0.22**	**< 0.05**	Y
	Fall	**0.08**	**0.10**	**< 0.05**	Y
Aerosol optical depth (500 nm)	Winter	**0.06**	**0.07**	**< 0.05**	N
	Spring	**0.10**	**0.12**	**< 0.05**	Y
	Summer	**0.14**	**0.18**	**< 0.05**	Y
	Fall	**0.07**	**0.09**	**< 0.05**	N
Aerosol optical depth (675 nm)	Winter	**0.04**	**0.05**	**< 0.05**	N
	Spring	**0.06**	**0.08**	**< 0.05**	Y
	Summer	**0.08**	**0.11**	**< 0.05**	Y
	Fall	**0.04**	**0.06**	**< 0.05**	N
Aerosol optical depth (870 nm)	Winter	**0.03**	**0.03**	**< 0.05**	N
	Spring	**0.04**	**0.06**	**< 0.05**	N
	Summer	**0.06**	**0.08**	**< 0.05**	Y
	Fall	**0.03**	**0.04**	**< 0.05**	N

Table S2. Total days binned as "clear sky" or "cloudy" using the AERONET quality assurance algorithm method for each season before matching with IMPROVE dates at Bondville from 2010 - 2019 and related days with GOES cloud top temperature observations for 2017.

	AERONET (2010 - 2019)			*GOES (2017)*		
	Clear	Cloudy	Total	Clear	Cloudy	Total
Winter	183	31	214	33	9	56
Spring	232	87	319	44	16	60
Summer	305	80	385	53	14	67
Fall	245	62	307	45	5	50

Table S3. Predominantly clear sky and cloudy medians and two-sided Mann-Whitney U test p values for meteorological variables of temperature, relative humidity, and planetary boundary layer height. For clarity, p values significantly less than 0.05 are listed as "< 0.05" and statistically significant seasons are bolded.

Parameter	Season	Clear Median	Cloudy Median	Mann-Whitney U test p value
Temperature (°C)	Winter	-1.3	4.0	0.505
	**Spring**	**11.5**	**20.1**	**< 0.05**
	Summer	24.1	24.8	0.324
	Fall	14.6	12.1	0.132
Relative Humidity (%)	Winter	64.7	64.2	0.915
	Spring	53.2	57.0	0.106
	Summer	67.1	68.2	0.561
	Fall	58.6	58.6	0.439
Planetary Boundary Layer Height (km)	Winter	0.49	0.51	0.961
	**Spring**	**0.66**	**0.57**	**< 0.05**
	Summer	**0.60**	**0.54**	**< 0.05**
	Fall	0.55	0.41	0.055

Table S4. Predominantly clear sky and cloudy distributions two-sided Mann-Whitney U test p values for IMPROVE-measured $PM_{2.5}$ chemical species and aerosol liquid water estimates. For clarity, p values significantly less than 0.05 are listed as "< 0.05" and statistically significant seasons are bolded.

Parameter	Season	Clear Median	Cloudy Median	Mann-Whitney U test p value
Inorganic Aerosol Liquid Water ($\mu g\ m^{-3}$)	Winter	1.60	1.14	0.329
	**Spring**	**1.10**	**1.97**	**< 0.05**
	Summer	2.19	2.81	0.093
	Fall	1.05	1.27	0.183
Organic Aerosol Liquid Water ($\mu g\ m^{-3}$)	Winter	0.58	1.29	0.898
	**Spring**	**0.34**	**0.62**	**< 0.05**
	Summer	1.42	1.86	0.116
	Fall	0.62	0.73	0.235
Nitrate ($\mu g\ m^{-3}$)	Winter	2.52	1.60	0.650
	Spring	0.86	0.85	0.621
	Summer	0.30	0.33	0.443
	Fall	0.45	0.84	0.052
Sulfate ($\mu g\ m^{-3}$)	Winter	1.10	0.92	0.301
	**Spring**	**0.97**	**1.32**	**< 0.05**
	Summer	1.27	1.72	0.113
	Fall	0.45	0.90	0.599
Organic Mass ($\mu g\ m^{-3}$)	Winter	1.55	1.77	0.961
	**Spring**	**1.31**	**1.99**	**< 0.05**
	Summer	3.13	3.55	0.110
	Fall	2.20	2.52	0.536

Potassium (μg m^{-3})	Winter	0.032	0.038	0.595
	**Spring**	**0.032**	**0.041**	**< 0.05**
	Summer	0.031	0.031	0.387
	Fall	0.036	0.037	0.496
Chloride (μg m^{-3})	Winter	0.058	0.038	0.258
	Spring	0.020	0.026	0.093
	Summer	0.016	0.016	0.425
	Fall	0.020	0.016	0.546
Calcium (μg m^{-3})	Winter	0.024	0.033	0.602
	Spring	0.035	0.031	0.907
	Summer	0.052	0.041	0.743
	Fall	0.066	0.058	0.193
Magnesium (μg m^{-3})	Winter	0.007	0.006	0.549
	Spring	0.012	0.017	0.073
	Summer	0.014	0.007	0.112
	Fall	0.013	0.007	0.256
Sodium (μg m^{-3})	Winter	0.020	0.018	0.816
	Spring	0.012	0.014	0.149
	Summer	0.010	0.013	0.249
	Fall	0.010	0.006	0.300
$PM_{2.5}$ Dry Mass + Aerosol Liquid Water (μg m^{-3})	Winter	10.20	10.20	0.716
	Spring	**6.22**	**10.47**	**< 0.05**
	Summer	10.30	13.40	0.083
	Fall	7.18	8.81	0.415

AUTHOR INFORMATION

Corresponding Author

*E-mail: agcarlto@uci.edu
